# Novel genetic variant of *HPS1* gene in Hermansky-Pudlak syndrome with fulminant progression of pulmonary fibrosis: a case report

**DOI:** 10.1186/s12890-019-0941-4

**Published:** 2019-10-16

**Authors:** Martina Doubková, Jakub Trizuljak, Zuzana Vrzalová, Anna Hrazdirová, Ivona Blaháková, Lenka Radová, Šárka Pospíšilová, Michael Doubek

**Affiliations:** 1 Department of Pulmonary Diseases and Tuberculosis, Masaryk University, Faculty of Medicine and University Hospital, Brno, Czech Republic; 2 Department of Internal Medicine, Hematology and Oncology, University Hospital and Faculty of Medicine, Jihlavská 20, 625 00 Brno, Czech Republic; 3 Central European Institute of Technology, Masaryk University, Brno, Czech Republic

**Keywords:** Exome sequencing, Hermansky-Pudlak syndrome, Pulmonary fibrosis

## Abstract

**Background:**

Hermansky-Pudlak syndrome (HPS) is an autosomal recessive disorder that is associated with oculocutaneous albinism, bleeding diathesis, granulomatous colitis, and highly penetrant pulmonary fibrosis in some subtypes. Homozygous or compound heterozygous pathological variants in *HPS1, HPS3, HPS4,* and several other genes lead to clinical manifestation of the disease.

**Case presentation:**

A 57-year-old female was admitted with congenital oculocutaneous albinism, thrombocytopathy and late-onset accelerated pulmonary fibrosis (first symptoms from age 50 onwards). Chest high-resolution computed tomography identified thickening of peribronchovascular interstitium, bronchiectasis, reticulations, honeycombing, ground glass opacities and lung parenchyma consolidations. HPS was clinically suspected. We performed whole exome sequencing (WES), a form of massive parallel sequencing, of proband-parents trio. Whole exome libraries were processed using KAPA Hyper Prep Kit, SeqCap EZ MedExome Enrichment Kit and HyperCap Bead Kit according to the SeqCap EZ HyperCap Workflow. The paired-end 2 × 75 bp sequencing was performed on the Illumina NextSeq 500 Sequencer (Illumina Inc., USA). Furthermore, obtained variants by WES were evaluated using a virtual panel of genes: *HPS1, AP3B1, HPS3, HPS4, HPS5, HPS6, DTNBP1, BLOC1S3,* and *PLDN*. We identified a compound heterozygous genotype in *HPS1* gene in the proband. We identified a pathogenic frameshift variant c.1189delC; p.(Gln397Serfs*2), resulting in a premature stop codon. This variant has been previously associated with HPS. Furthermore, we characterized previously undescribed nonsense variant c.1507C > T; p.(Gln503*), resulting in a premature stop codon and mRNA degradation through nonsense-mediated decay. Sanger sequencing validated the presence of both variants and simultaneously confirmed the heterozygous carrier status of parents. Unfortunately, the patient died due to fulminant progression of pulmonary fibrosis 2 months after diagnostics.

**Conclusions:**

Compound heterozygous mutations in *HPS1* in the proband lead to disruption of *HPS1* gene and clinical manifestation of HPS with severe pulmonary fibrosis. This case illustrates the need to consider HPS in differential diagnostics of pulmonary fibrosis. Pulmonary fibrosis is a common cause of death in HPS patients. Earlier diagnosis may enable better treatment for these patients.

## Background

Hermansky-Pudlak syndrome (HPS) is an autosomal recessive disorder associated with oculocutaneous albinism (or some degree of hypopigmentation), decreased visual activity generally accompanied by horizontal nystagmus, bleeding diathesis, granulomatous colitis, and highly penetrant pulmonary fibrosis in some subtypes. The HPS spectrum includes ten disorders (HPS-1 to HPS-10). Homozygous or compound heterozygous mutations in *HPS1, HPS3, HPS4,* and several other genes lead to clinical manifestation of the disease [1–3]. The disease was described by two Czech hematologists František Heřmanský and Pavel Pudlák in 1959 [4].

## Case presentation

**Fig. 1:**
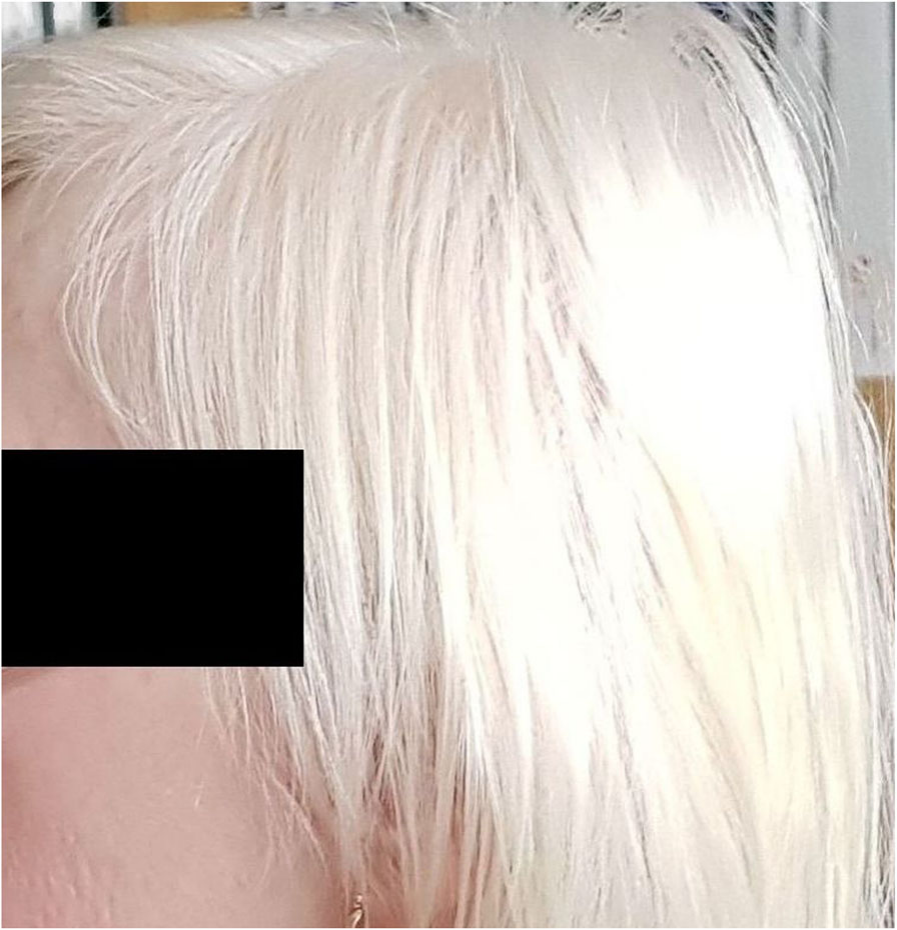
Albinism in the Hermansky-Pudlak syndrome patient

A 57-year-old Caucasian female (proband), teacher, with oculocutaneous albinism (Fig. 1) was admitted for dry cough and rapid worsening of dyspnea. A thorough analysis of the medical history revealed that the patient had eye problems since childhood and that from the age of 45, her vision was significantly worse. Furthermore, it was found that she had several episodes of prolonged bleeding: after appendectomy, after minor injuries (including hemartros) and after childbirth. At the age of 50, she was examined by a hematologist. Platelet aggregation was performed, showing slightly prolonged PFA-100 time in the presence of collagen/ADP. No definite conclusion has been made regarding this finding. The first mild lung problems occurred at the age of 53. She was followed with diagnosis of bronchial asthma by a regional pneumologist. There was no family history of these symptoms. She was a non-smoker.

Physical examination revealed clubbing fingers and bilateral end-inspiratory fine crackles in the lower and middle lung areas. The posteroanterior chest X-ray showed bilateral diffuse reticular opacities.

**Fig. 2:**
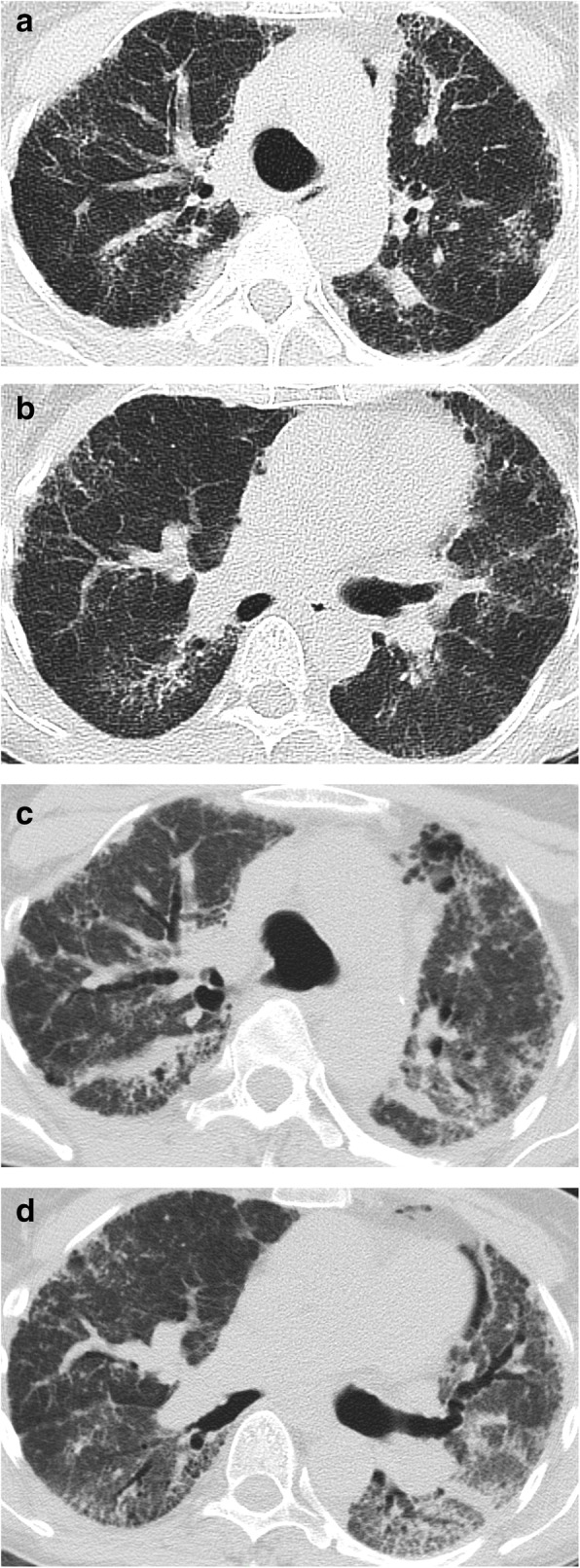
High-resolution computed tomography (axial plane) of the chest showing worsening of lung fibrosis with thickening of peribronchovascular interstitium, bronchiectasis, reticulations, honeycombing, ground glass opacities and lung parenchyma consolidations. Initial examination (**a**, **b**) and three-month follow-up (**c**, **d**)

**Fig. 3:**
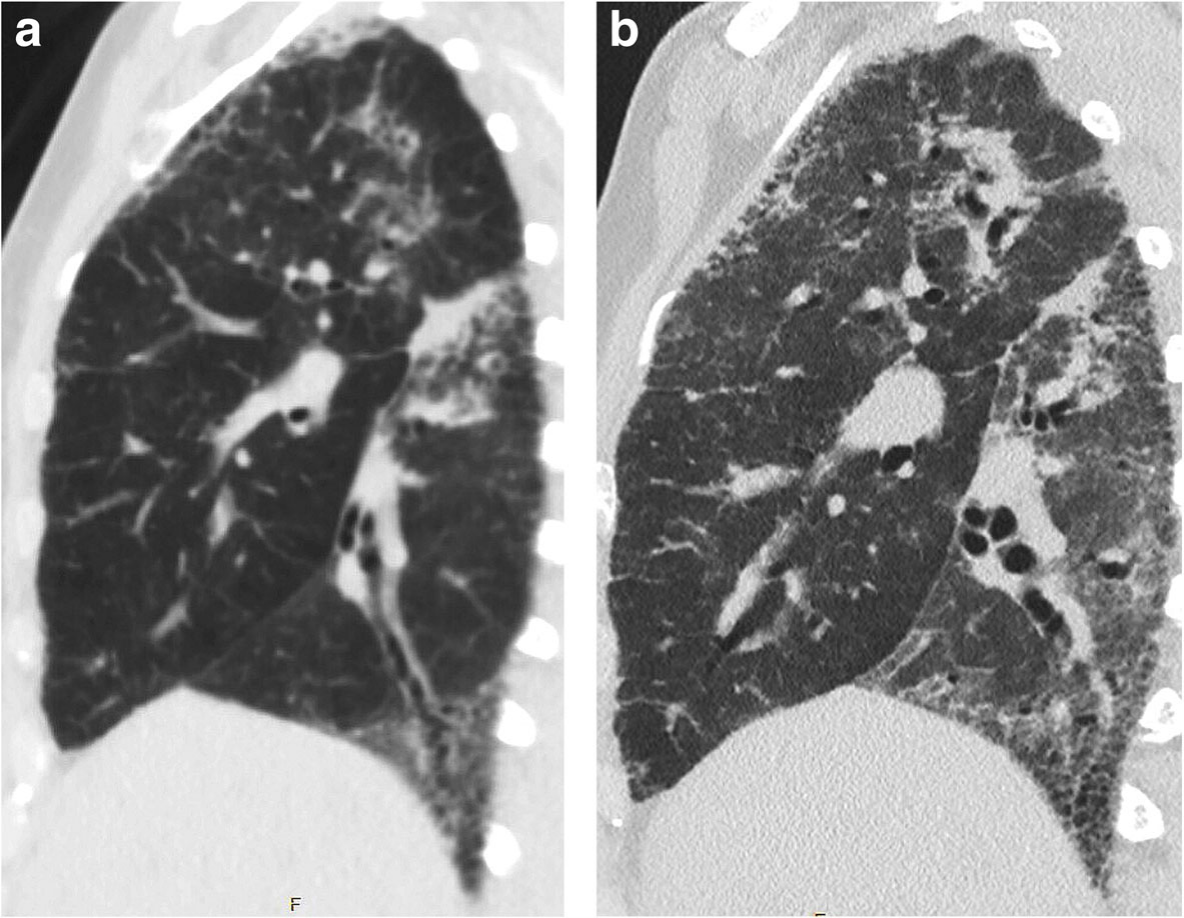
High-resolution computed tomography (sagital plane) of the chest showing worsening of lung fibrosis with thickening of peribronchovascular interstitium, bronchiectasis, reticulations, honeycombing, ground glass opacities and lung parenchyma consolidations. Initial examination (**a**) and three-month follow-up (**b**)

High-resolution computed tomography (HRCT) of the chest identified thickening of the peribronchovascular interstitium, bronchiectasis, reticulations, honeycombing, ground glass opacities and lung parenchyma consolidations. A comparison of HRCT images performed at 3-month intervals showed fulminant progression of pulmonary involvement (Figs. 2 and 3).

Pulmonary function testing revealed severe restrictive ventilation impairment and a severe decline of diffusing capacity of the lung for carbon monoxide (DLco 20%). Arterial blood gas analysis showed hypoxemia (p02 7 kPa). Moderate pulmonary hypertension was found. Blood count, serum biochemistry, and immunologic parameters were normal.

**Fig. 4:**
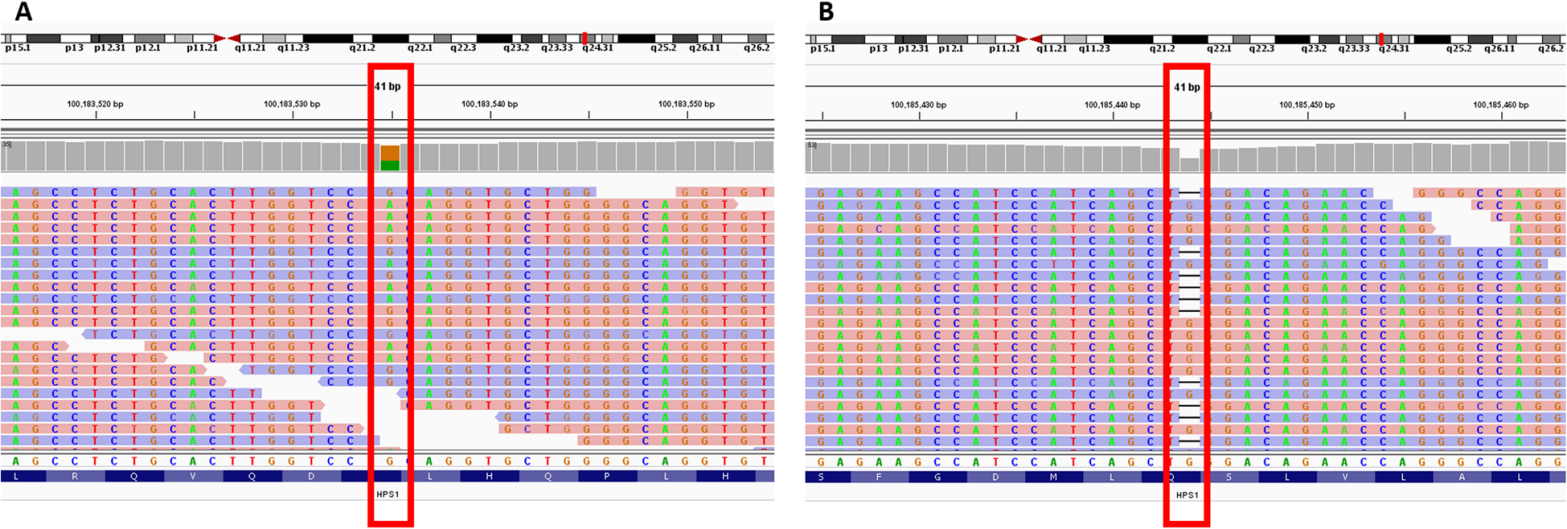
Visualization of c.1507C > T variant (g.100183535C > T) (**a**) and of c.1189delC (g.100185444) variant (**b**) by Intergrative Genomics Viewer. Variants are marked by red frames. Forward sequencing reads are in blue; reverse sequencing reads are in pink

Based on these findings, HPS was suspected. The negative family history of symptoms suggested an autosomal-recessive mode of inheritance. Therefore, whole exome sequencing (a form of massive parallel sequencing) of the proband-parent trio was carried out. Samples of peripheral blood were collected and processed for genomic DNA isolation using MagCore® Genomic DNA Whole Blood Kit (RBC Bioscience). Whole exome libraries were processed using KAPA Hyper Prep Kit, SeqCap EZ MedExome Enrichment Kit and HyperCap Bead Kit (Roche, USA) according to the SeqCap EZ HyperCap Workflow v2.1 following the recommended protocols. Paired-end 2 × 75 bp sequencing was performed on the Illumina NextSeq 500 Sequencer (Illumina Inc., USA). The raw sequencing reads were aligned to the GRCh37 (hg19) human reference genome using the BWA-mem algorithm, version 0.7.15, PCR duplicates were identified with the MarkDuplicates tool from Picard. Germline single nucleotide variants (SNV) and indels were detected by the GATK HaplotypeCaller, version 3.7. Annotation of obtained variants/indels was performed with Annovar. Furthermore, the processed variants/indels were matched to the virtual panel of genes including *HPS1, AP3B1, HPS3, HPS4, HPS5, HPS6, DTNBP1, BLOC1S3,* and *PLDN*. The virtual panel was created based on the literature review [1–3]. Exome sequencing identified a compound heterozygous genotype in the *HPS1* gene (NM_000195.3) in the proband: 1) pathogenic frameshift variant c.1189delC; p.(Gln397Serfs*2), resulting in a premature stop codon, associated with HPS; and 2) previously undescribed nonsense variant, c.1507C > T; p.(Gln503*), resulting in a premature stop codon, implying a loss of 197 amino acids or more likely, nonsense-mediated decay of the mRNA degradation (Fig. 4).

The coverage range of the novel variant c.1507C > T in the proband was 26 reads and variant allele frequency range 38.5%.

**Fig. 5:**
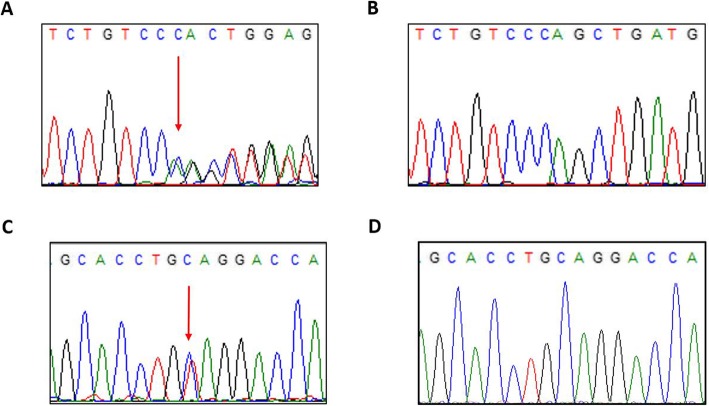
Sanger sequencing results of c.1189delC variant (**a**) compared with wild-type (**b**) and of c.1507C > T variant (**c**) compared with wild-type (**d**)

Subsequently, the diagnosis has been verified using PCR and Sanger sequencing of the amplicons in the proband, and also the presence of heterozygous carrier status of parents (Fig. 5). Primers were designed for exons 13 and 15, respectively (13F-primer: CTTAGGGTTGGCACGTCTTC, 13R-primer: TGGGTCTCACCTGAATCTCC; 15F-primer: TTCTGCTGTAATGCCCTCCT, 15R-primer: GAAGTCCTTCCAGTCCGTCA). PCR was performed with the annealing temperature 60 °C using Q5 High-Fidelity DNA Polymerase (New England Biolabs Inc., England) according to the manufacturer’s protocol. PCR products were purified using the Qiaquick PCR purification kit (QIAGEN, Germany). Capillary sequencing was performed using BigDye-terminator chemistry on 3500 Genetic Analyzer (Applied Biosystems, USA).

We could not analyze the structural effect of the variant p.(Gln503*) “in silico” because the crystal structure was not available. However, given the type of “nonsense” variant, we can assume that the novel variant including the nucleotide change C > T at the position 1507 leads to a shortened protein which most likely results in misfolding of the protein and impaired function.

Treatment with corticosteroids, started before HPS diagnosis was confirmed, had no effect on pulmonary functions. Therefore, lung transplantation began to be prepared. Unfortunately, 2 months after HPS diagnostics, the patient died due to ongoing fulminant lung fibrotization.

## Discussion and conclusions

HPS is rare and heterogenous autosomal recessive disease characterized by abnormalities in both lysosomes and lysosome-related organelles. The disease is rare in Caucasians but is the most prevalent cause of albinism in Puerto Rico [5]. Ten subtypes of HPS (HPS-1 to HPS-10) have been reported; three HPS subtypes are associated with fibrotic lung disease: HPS-1, HPS-2, and HPS-4. HPS can be caused by at least nine genes: *HPS1, AP3B1, HPS3, HPS4, HPS5, HPS6, DTNBP1, BLOC1S3,* and *PLDN*.

To date, 61 variants in the *HPS1* gene have been reported as disease causing or likely disease causing according to the Human Gene Mutation Database (Table 1) [7]. The most common pathogenic variants of *HPS1* gene are nonsense/missense or small deletions. Pulmonary fibrosis is seen in approximately one half of carriers of *HPS1* and *HPS4* gene mutations [2, 3, 6]. However, other *HPS1* gene variants are associated with milder symptoms like albinism, nystagmus, hypopigmentation, foveal hypoplasia or absent nails [7].

**Table 1:** Phenotypes associated with various *HPS1* gene variants according to the Human Gene Mutation Database [6]

Variant type	Total number of described variants	Reported phenotype
Missense/nonsense	22	Hermansky-Pudlak syndrome; albinism; nystagmus; hypopigmentation; foveal hypoplasia; absent nails
Splicing substitutions	8	Hermansky-Pudlak syndrome
Small deletions	17	Hermansky-Pudlak syndrome
Small insertions/duplications	7	Hermansky-Pudlak syndrome
Small indels	1	Hermansky-Pudlak syndrome
Gross deletions	5	Hermansky-Pudlak syndrome
Complete rearrangements	1	Hermansky-Pudlak syndrome

It has been described that the majority of HPS patients are compound heterozygotes [8]. Our proband was also a compound heterozygote carrying previously described frameshift variant c.1189delC and novel nonsense variant c.1507C > T. Theunissen et al. reported a patient who was compound heterozygote with the same c.1189delC variant as in our case and different nonsense variant c.517C > T. This patient suffered from an oculocutaneous albinism and “multisystemic disease” since childhood [9]. Hermos et al. described four novel *HPS1* variants in non-Puerto Rican patients suffered from HPS, where small deletions of nucleotide C (c.561delC) and nucleotide A (c.1581delA) of *HPS1* gene producing no RNA have been found. One of these patients developed pulmonary fibrosis, two patients had granulomatous colitis [10]. So far eight disease-causing variants of the nonsense type have been described. In one Pakistani family, a nonsense variant p.(Gln686*) of the *HPS1* gene was segregating with the HPS phenotype. An absence of pulmonary fibrosis in these affected individuals might be due to their relatively young age [11]. On the other hand, Abouelhoda et al. detected nonsense *HPS1* variant in exon 14 associated with absent nails only [12].

HPS subtypes with lung fibrosis have a poorer prognosis compared with other types of HPS. Clinical manifestations of HPS-associated pulmonary fibrosis occur usually in the fourth or fifth decade of life [1–3].

Radiological findings of HPS pulmonary fibrosis are variable: reticular opacities, septal and pleural thickening, bronchiectasis, ground-glass opacities, loss of lung volume, or honeycombing. Predominant radiographic findings are found in the lung periphery and progress toward the central portion of the lung [13].

The average life expectancy of patients with HPS is 40–50 years. Pulmonary fibrosis is a common cause of death in HPS patients [13, 14]. There is no known curative therapy for HPS. Corticosteroids are not effective. Pirfenidone, an antifibrotic agent, has been shown to slow fibrosis progression, but only in patients who have well-preserved residual lung volume [3, 7]. Thus, lung transplantation remains the only means of prolonging the survival of HPS patients with advanced pulmonary fibrosis [15]. A potential contraindication to performing lung transplant is thrombocytopathy associated with HPS. This condition can be managed by intravenous desmopressin administration and platelet transfusions [16].

Compound heterozygous mutations in *HPS1* in our proband led to the disruption of *HPS1* gene and clinical manifestation of HPS with severe pulmonary fibrosis. This case illustrates the need to consider HPS in differential diagnostics of pulmonary fibrosis. Earlier diagnosis of HPS may aid the timing of lung transplantation. Our case also shows that progression of HPS-associated fibrosis may be fulminant. Therefore, an indication for lung transplantation cannot be delayed.

## Data Availability

The datasets used and/or analysed during the current study are available from the corresponding author on reasonable request.

## Abbreviations

DLco: Diffusing capacity of the lung for carbon monoxide
HPS: Hermansky-Pudlak syndrome
HRCT: High resolution computed tomography
NGS: Next-generation sequencing
SNV: Single nucleotide variants

## References

[1] Huizing M, Helip-Wooley A, Westbroek W, Gunay-Aygun M, Gahl WA (2008). Disorders of lysosome-related organelle biogenesis: clinical and molecular genetics. Annu Rev Genomics Hum Genet.

[2] Gahl WA, Brantly M, Kaiser-Kupfer MI, Iwata F, Hazelwood S, Shotelersuk V (1998). Genetic defects and clinical characteristics of patients with a form of oculocutaneous albinism (Hermansky-Pudlak syndrome). N Engl J Med.

[3] McElvaney OJ, Huizing M, Gahl WA, O’Donovan P, Horan D, Logan PM (2018). Hermansky-Pudlak syndrome with a novel genetic variant in HPS1 and subsequent accelerated pulmonary fibrosis: significance for phenocopy diseases. Thorax.

[4] Hermansky F, Pudlak P (1959). Albinism associated with hemorrhagic diathesis and unusual pigmented reticular cells in the bone marrow: report of two cases with histochemical studies. Blood.

[5] Witkop CJ, Nuñez Babcock M, Rao GH, Gaudier F, Summers CG, Shanahan F (1990). Albinism and Hermansky-Pudlak syndrome in Puerto Rico. Bol Asoc Med P R.

[6] Gahl WA, Brantly M, Troendle J, Avila NA, Padua A, Montalvo C (2002). Effect of pirfenidone on the pulmonary fibrosis of Hermansky-Pudlak syndrome. Mol Genet Metab.

[7] Stenson PD, Mort M, Ball EV, Evans K, Hazden M, Heywood S (2017). The human gene mutation database: towards a comprehensive repository of inherited mutation data for medical research, genetic diagnosis and next-generation sequencing studies. Hum Genet.

[8] Wei A, Yuan Y, Bai D, Ma J, Hao Z, Zhang Y (2016). NGS-based 100-gene panel of hypopigmentationidentifies mutations in Chinese Hermansky–Pudlak syndrome patients. Pigment Cell Melanoma Res.

[9] Theunissen TEJ, Sallevelt SCEH, Hellebrekers DMEI, de Koning B, Hendrickx ATM, van den Bosch BJC (2017). Rapid resolution of blended or composite multigenic disease in infants by whole-exome sequencing. J Pediatr.

[10] Hermos CR, Huizing M, Kaiser-Kupfer MI, Gahl WA (2002). Hermansky-Pudlak syndrome type 1: gene organization, novel mutations, and clinical-molecular review of non-Puerto Rican cases. Hum Mutat.

[11] Yousaf S, Shahzad M, Tasleem K, Sheikh SA, Tariq N, Sabbir AS (2016). Identification and clinical characterization of Hermansky-Pudlak syndrome alleles in the Pakistani population. Pigment Cell Melanoma Res.

[12] Abouelhoda M, Sobahy T, El-Kalioby M, Patel N, Shamseldin H, Monies D (2016). Clinical genomics can facilitate countrywide estimation of autosomal recessive disease burden. Genet Med.

[13] Vicary GW, Vergne Y, Santiago-Cornier A, Young LR, Roman J (2016). Pulmonary fibrosis in Hermansky-Pudlak syndrome. Ann Am Thorac Soc.

[14] Brantly M, Avila NA, Shotelersuk V, Lucero C, Huizing M, Gahl WA (2000). Pulmonary function and high-resolution CT findings in patients with an inherited form of pulmonary fibrosis, Hermansky-Pudlak syndrome, due to mutations in HPS-1. Chest.

[15] Lederer DJ, Kawut SM, Sonett JR, Vakiani E, Seward SL, White JG (2005). Successful bilateral transplantation for pulmonary fibrosis associated with the Hermansky-Pudlak syndrome. J Heart Lung Transplant.

[16] El-Chemaly S, O'Brien KJ, Nathan SD, Weinhouse GL, Goldberg HJ, Connors JM (2018). Clinical management and outcomes of patients with Hermansky-Pudlak syndrome pulmonary fibrosis evaluated for lung transplantation. PLoS One.

